# Probing collective terahertz vibrations of a hydrogen-bonded water network at buried electrochemical interfaces†

**DOI:** 10.1039/d3sc01734f

**Published:** 2023-05-15

**Authors:** Taichi Isogai, Masayuki Uranagase, Kenta Motobayashi, Shuji Ogata, Katsuyoshi Ikeda

**Affiliations:** a Department of Physical Science and Engineering, Nagoya Institute of Technology Nagoya 466-8555 Japan kikeda@nitech.ac.jp; b Frontier Research Institute for Materials Science (FRIMS), Nagoya Institute of Technology Nagoya 466-8555 Japan

## Abstract

The exceptional properties of liquid water such as thermodynamic, physical, and dielectric anomalies originate mostly from the hydrogen-bond networks of water molecules. The structural and dynamic properties of the hydrogen-bond networks have a significant impact on many biological and chemical processes in aqueous systems. In particular, the properties of interfacial water molecules with termination of the network at a solid surface are crucial to understanding the role of water in heterogeneous reactions. However, direct monitoring of the dynamics of hydrogen-bonded interfacial water molecules has been limited because of the lack of a suitable surface-selective spectroscopic means in the terahertz (THz) frequency range where collective vibrations of water exist. Here we show that hydrogen-bond vibrations below 9 THz can be measured *in situ* at an electrochemical interface, which is buried between two THz-opaque media, by using a density of states format of surface-enhanced inelastic light scattering spectra. The interpretation of the obtained spectra over the range 0.3–6 THz indicates that the negatively charged surface accelerates collective translational motions of water molecules in the lateral direction with the increase of hydrogen-bond defects. Alternatively, the positively charged surface results in suppression of lateral mobility. This work gives a new perspective on *in situ* spectroscopic investigations in heterogeneous reactions.

## Abstract 2

In a density of states format of the frequency-extended surface-enhanced Raman spectrum, potential-induced variations of the lifetime of hydrogen bonds and their dynamic behavior were observed at aqueous solution/Au interfaces.

## Graphical abstract

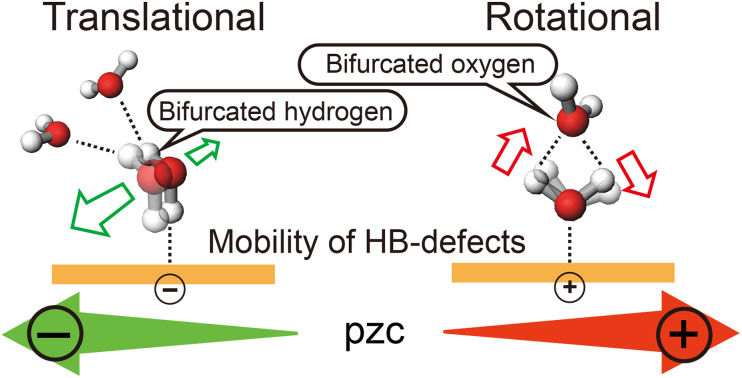

## Introduction

The interaction of water with surfaces is a topic of great interest in a wide variety of scientific fields.^1–3^ In particular, aqueous electrochemical interfaces are central to heterogeneous catalysis and emerging technologies for energy conversion. Water molecules at such charged interfaces are known to exhibit significantly different properties than water in the bulk liquid. It is therefore imperative to characterize the inhomogeneous nature of the electrochemical interface, *i.e.*, the electric double layer. Surface-selective vibrational spectroscopy has provided many insights into the properties of aqueous electrochemical interfaces based on the analysis of the O–H stretching band *ν*(OH) at ∼3400 cm^−1^ (∼100 THz) and H–O–H bending band *δ*(HOH) at ∼1600 cm^−1^ (48 THz).^4–14^ However, these intramolecular vibration modes of individual water molecules provide only limited information about the dynamic properties of the hydrogen-bond networks.^15^ Indeed, most of the spectroscopic studies on interfacial water have focused on rather static characteristics such as orientation of water molecules. Given that the peculiar properties of liquid water originate mostly from the hydrogen-bond networks,^16^ it is crucial to be able to observe the collective intermolecular vibrations of interfacial water below 300 cm^−1^ (9 THz) to gain deeper insights into the dynamic behaviour of liquid water. For the bulk liquid, such collective THz vibrations can be measured using far-IR absorption, neutron scattering, and low-frequency Raman scattering.^17–21^ Accordingly, the bulk behaviour of water in the THz range is quite actively studied from both experimental and theoretical points of view.^22–26^ On the other hand, the interfacial behaviour of water in the THz range remains unexplored in the experimental field. Electrochemical interfaces are buried between two THz-opaque media: metallic electrode and aqueous solution. Therefore, it is still a challenge to probe the THz responses of the water molecules within the electric double layer.^27^ Among various surface-selective vibrational spectroscopies, surface-enhanced Raman scattering (SERS) is potentially capable of detecting THz vibrations at such a buried interface. However, THz responses in SERS spectra are invariably concealed by a large spectral background generated from plasmonic metal surfaces, which is problematic in analysing THz-vibrations.^28,29^ Time-domain Raman spectroscopy may be one of the options to obtain THz-vibrations without the use of plasmon resonances.^30,31^ However, there is a serious drawback of this technique to obtain one spectrum for the THz region in real time. So far, no THz-vibrational spectroscopy has been applied to direct monitoring of the hydrogen-bond networks of interfacial water.

Here, we overcome the challenge by using a density of states format of frequency-extended SERS spectra. This technique is based on the fact that the spectral background in SERS has recently been ascribed to plasmon-enhanced electronic Raman scattering in the metal.^32–37^ That is, the vibrational and electronic signals in the power spectrum of SERS are connected with vibrational and electronic Raman susceptibilities, respectively, through the Bose–Einstein thermal factor, frequency factor, and the Purcell factor.^32–34^ In this work, we demonstrate that the reduction of these factors from the measured spectra finds its greatest application in analysing THz responses of interfacial water molecules. Potential-induced changes of dynamic behaviour of water on gold surfaces are compared among various electrolyte solutions, and the result clearly shows that the dynamic behaviour of interfacial water molecules is indeed different from that of bulk water. The distinctive interfacial water dynamics is caused by the local defects of hydrogen-bond networks, which originated from surface charge-induced orientation of interfacial water.

## Methods

### Electrochemical and spectroscopic measurements

A SERS-active Au electrode was obtained using electrochemical surface roughening of Au in 0.1 M KCl aqueous solution. The roughened surface was carefully cleaned to remove Cl^−^ anions, which was confirmed by the disappearance of the *ν*Au-Cl^−^ peak at 265 cm^−1^ in the SERS spectrum. The electrochemical SERS spectra for the aqueous solution/Au interface were measured in a three-electrode cell filled with Ar-bubbled aqueous solutions with different electrolytes. The electrochemical potentials, measured against the Ag/AgCl, were converted to the potentials *vs.* the reversible hydrogen electrode (RHE) to compare the pH dependence of SERS spectra; during the electrochemical SERS measurement, the possibility of Cl^−^ adsorption on the Au surface was excluded by the absence of the *ν*Au-Cl^−^ peak. The respective potentials of zero charge (pzc) for the different solution/Au interfaces were determined by measuring the double-layer capacities at the SERS-active Au surfaces.^29^

SERS spectra were recorded using a home-built inverted microscope Raman system with an objective (40×, 0.6 N.A.).^28^ A He–Ne laser radiation of 632.8 nm was further monochromatized using an ultra-narrow band laser-line filter with a bandwidth of less than 0.4 nm FWHM (reflecting volume Bragg grating (VBG) line filter, OptiGrate Corp).^38^ The excitation laser power was typically less than 0.02 mW at the focal point, which is low enough to avoid local heating of Au surfaces.^32^ The backscattered Raman signals from the sample were monitored using a single-stage spectrometer with a CCD detector (IsoPlane & PIXIS 100BR eX-I, Princeton Instruments) after Rayleigh scattering was removed by ultra-narrow band VBG notch filters (OptiGrate Corp).

The measured SERS spectra were converted to SERS susceptibility (*χ*′′_SERS_) by reducing the Bose–Einstein thermal factor, frequency factor, and the Purcell factor, according to the procedure reported previously,^32–34^ so that low-frequency vibrations were unveiled from the spectral background generated by the electronic Raman scattering. To obtain the Purcell factor, the normal Raman spectrum for a SERS-inactive smooth Au surface was used as a reference. Normal Raman spectra for bulk solutions were also converted to vibrational Raman susceptibility (*χ*′′_VRS_) by reducing the thermal and frequency factors. For details, see ESI.†

### Computational method

Classical molecular dynamics (MD) simulations of water molecules were performed using the TIP4P/2005 model,^39^ which represents each water molecule as a rigid body. For simulation of the electrochemical interfaces, 4000 water molecules were inserted between two Au slabs located parallel to each other at a distance of 7.6 nm. To illustrate the inhomogeneous nature of the electrochemical interface, the polarizable Lennard-Jones potential reported by Geada *et al.*^40^ was adopted for the Au atoms. A charged Au surface was prepared by adjusting the charge of the cores of the Au atoms on the surface. When one Au slab is positively charged, the other slab is negatively charged so that the entire system maintains charge neutrality. The short-range interactions between water and Au atoms were treated using the Lennard-Jones potential with the parameters obtained according to the Lorentz–Berthelot rule. The Coulomb potential was evaluated by the standard Ewald method. The system was first equilibrated for 1 ns under a canonical ensemble by maintaining the temperature at 300 K using the velocity rescaling method developed by Bussi *et al.*^41^ Subsequently, the simulation for data production was conducted for 1 ns under a microcanonical ensemble. For details, see ESI.†

## Results and discussion

### Reduction of SERS spectra

**Fig. 1:**
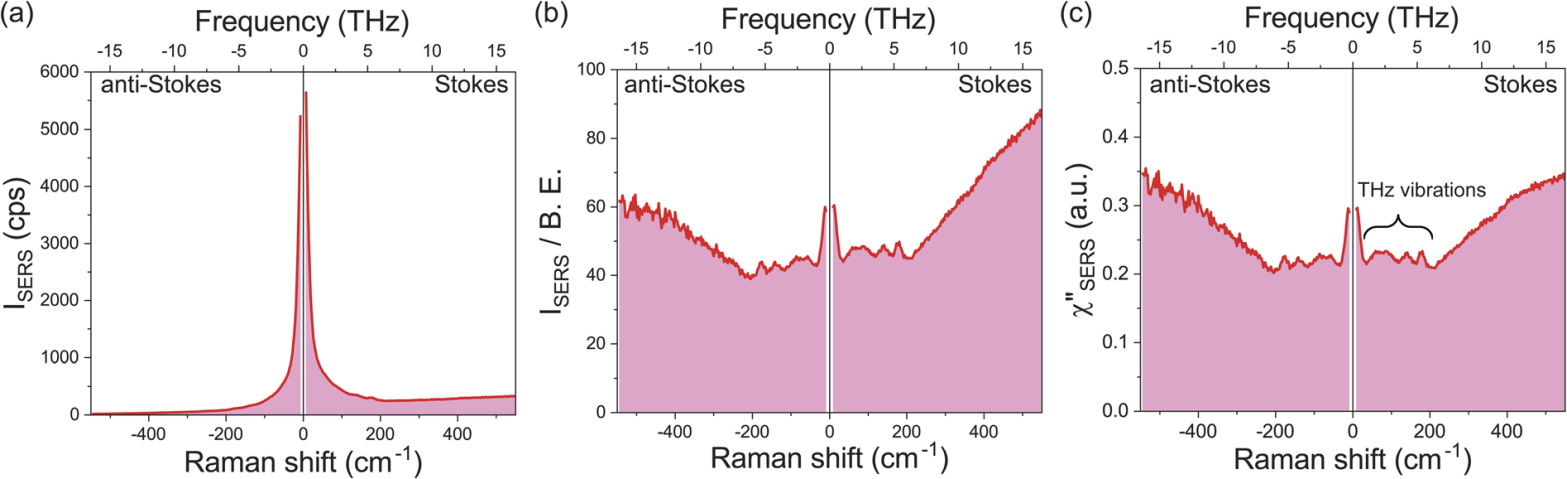
A raw SERS spectrum for the 0.1 M H_2_SO_4_/Au interface covering both Stokes and anti-Stokes branches, measured on an electrochemically roughened Au surface using 632.8 nm excitation. (b) Reduction of the Bose–Einstein thermal factor and frequency factor in (a). (c) Reduction of the Purcell factor in (b), which corresponds to the *χ*′′_SERS_ spectrum composed of the vibrational and electronic responses at the interface in a density of states format.

Fig. 1a shows a raw SERS spectrum for the 0.1 M H_2_SO_4_ aqueous solution/Au interface in the Stokes and anti-Stokes branches covering the low-frequency region above 10 cm^−1^ (0.3 THz). There is a steep increase of SERS intensities below 200 cm^−1^ (6 THz), which is typically seen in the SERS spectrum taken for plasmonic metal surfaces in aqueous solutions,^28,29,42^ and hence, THz-vibrational features are unrecognized in the raw spectrum. As reported previously,^32^ these SERS signals in the THz-shifted region can be dominantly ascribed to electronic SERS signals. Fig. 1b shows the spectrum of Fig. 1a divided by the Bose–Einstein distribution function at a local temperature of 300 K, in which small vibrational features below 200 cm^−1^ (6 THz) become recognizable on the huge background continuum. Fig. 1c shows the surface-enhanced Raman susceptibility (*χ*′′_SERS_) spectrum obtained from Fig. 1b by reducing the radiative Purcell factor,^43,44^ in which the symmetry of the response between the Stokes and anti-Stokes branches is confirmed in the wide frequency range for both the vibrational features and the background continuum. This symmetry means that the *χ*′′_SERS_ is correctly extracted from the measured SERS spectrum because the Stokes and anti-Stokes Raman processes are connected due to the time-reversal symmetry.^45^ It is noted that the *χ*′′_SERS_ treats a sum of vibrational and electronic Raman responses at the interface presented in a density of states format.^32–34^

This technique has a great advantage of rapid signal collection over the wide frequency range covering both intermolecular and intramolecular vibrations; it takes only a few seconds to obtain one spectrum, which enables *in situ* vibrational spectroscopic monitoring of electrochemical interfaces in the THz region. It is also emphasized that the local temperature of the sample under laser illumination, which is sometimes problematic in laser spectroscopies, can be quantitatively evaluated through the thermal factor used in this method.^32^ From the degree of the symmetry between the Stokes and anti-Stokes branches in the reduced spectra, we evaluated that all the data in this work were taken at the local temperature of 300 ± 2 K.

### THz responses of bulk and interfacial water molecules

**Fig. 2:**
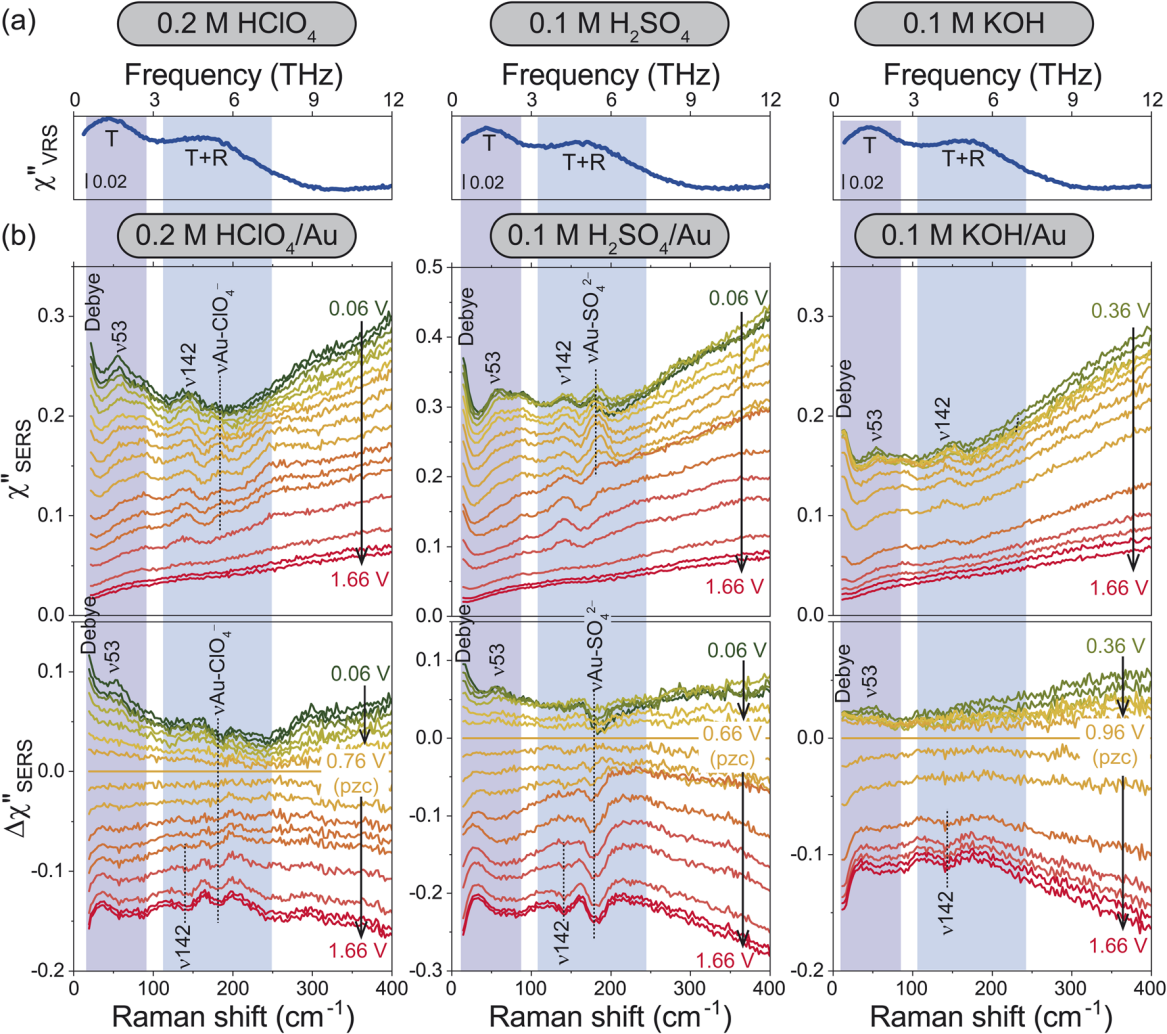
(a) *χ*′′_VRS_ spectra for bulk aqueous solutions containing different electrolytes. (b) Potential dependence of *χ*′′_SERS_ spectra for the respective electrolyte solutions on Au surfaces (upper panel) and their Δ*χ*′′_SERS_ spectra with respect to pzc for each solution (bottom).

For the bulk liquid phase of water, there are two intermolecular bands below 300 cm^−1^ (9 THz).^16^Fig. 2a shows Stokes branches of vibrational Raman susceptibility (*χ*′′_VRS_) spectra for bulk aqueous solutions with different electrolytes. In such dilute electrolyte solutions, the THz spectral features are nearly ion-independent; the collective translational mode, denoted as T, and the combination mode of collective translational and rotational motions, denoted as T + R, are found at 45 and 175 cm^−1^, respectively. (The consistency between this interpretation and the previous assignments^18–25^ is explained in the ESI.†)

For the interfacial water, the *χ*′′_SERS_ spectrum at Au/gas interfaces was first compared under different humidities, indicating that the collective modes of water molecules are indeed measurable using SERS (see Fig. S4†). Then, the *χ*′′_SERS_ spectra for the respective electrolyte solutions on rough Au surfaces were obtained under various electrochemical potential applications, as shown in the upper panel of Fig. 2b. (We have confirmed that the potential-induced change in the Purcell factor is negligible as shown in Fig. S3.†) To extract the potential-induced spectral variations, the difference spectra (Δ*χ*′′_SERS_) with respect to the potential of zero charge (pzc)^29^ for each solution are also shown in the bottom panel of the figure. The changes in the background intensities in Fig. 2b are caused by the potential-induced variation of the surface excess charge of Au.^29,46–48^ Except for anion-related vibrations such as the *ν*Au-ClO_4_^−^ at 181 cm^−1^ and the *ν*Au-SO_4_^2−^ at 185 cm^−1^,^49,50^ the potential-induced variations were similarly observed in all the spectra. Importantly, these variations were reproducible and reversible in the double layer potential region.

In the frequency region of the T mode, there is a vibrational feature at 53 cm^−1^ (*ν*53), which increases in intensity towards negative potentials from the pzc. At the low frequency edge of the detectable range, an additional potential-dependent spectral component (denoted as Debye) is evolved on the negatively charged surface, which is probably related to the so-called fast Debye relaxation of water.^25,26^ In the frequency region of the T + R mode, the potential-induced spectral change was not so clearly observed; a small peak at 142 cm^−1^ (*ν*142) was less-potential dependent until the surface oxide was formed, which was confirmed by the evolution of *ν*Au-O at around 560 cm^−1^ in Fig. S2 and S3† (the detailed assignment of the peak is unclear at the present stage). In addition, there seems to be a very broad potential-dependent feature in the Δ*χ*′′_SERS_ spectra. That is, the T + R mode for the interfacial water molecules is likely to decrease in intensity towards the negative potentials while the T mode for the interfacial water molecules increases in intensity. Such correlation between the THz modes of bulk water and the potential-induced changes in the Δ*χ*′′_SERS_ strongly suggest that the observed THz-features are indeed associated with collective vibrations of interfacial water molecules; the ion-independent nature of Δ*χ*′′_SERS_ is not surprising in the case of dilute electrolyte solutions. Most of the interfacial water molecules do not directly interact with ions even in the electric double layer.

According to the previously reported temperature dependence of the THz response of bulk water,^19^ the T mode increases in intensity with decreasing the temperature. The fast Debye relaxation component also shows the temperature dependence. These are related to the lifetime of the hydrogen-bonds of bulk water.^22–26^ That is, the potential dependence observed at the electrochemical interfaces indicates that the collective motions of the hydrogen-bonded local structures are modified by the surface charge.

### MD simulations of interfacial water molecules

**Fig. 3:**
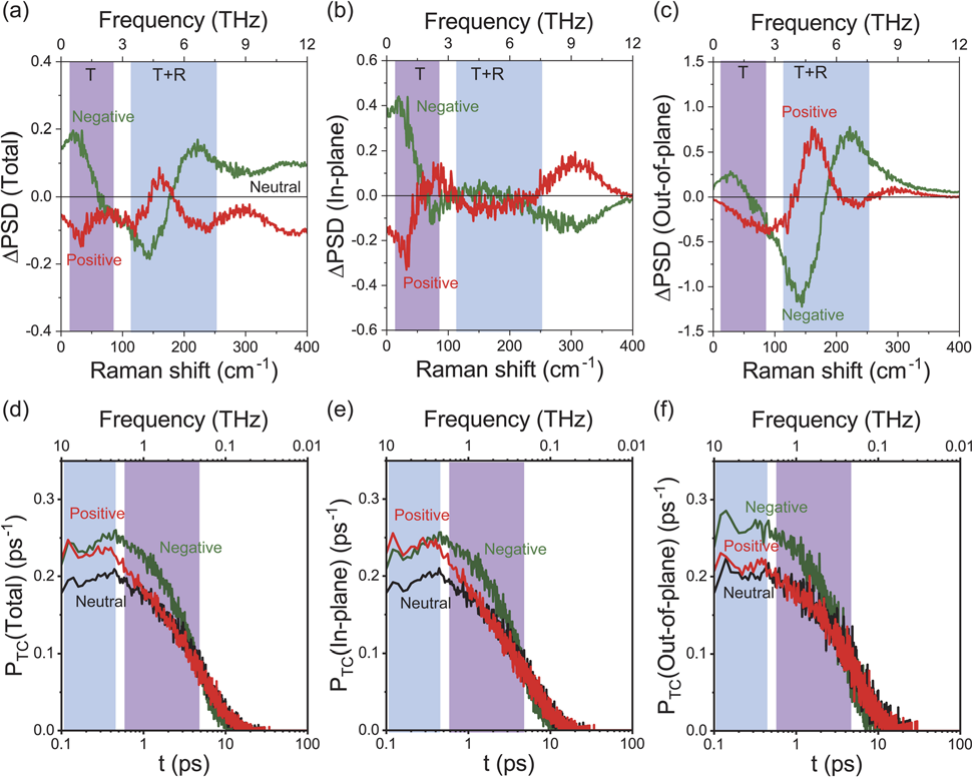
(a) ΔPSD of total velocities for the first-layer water molecules on negatively and positively charged Au(111) with respect to a neutral surface. (b) In-plane motions of the first-layer water network. (c) Out-of-plane motions of the first-layer water network. (d) The probability distribution of lifetime, *P*_TC_(*t*), for total hydrogen-bonds of the first-layer water molecules. (e) *P*_TC_(*t*) for in-plane hydrogen-bonds of the first-layer water molecules. (f) *P*_TC_(*t*) for out-of-plane hydrogen-bonds of the first-layer water molecules. The surface charge of 0.11C m^−2^ was applied in the simulation.

To obtain an in-depth understanding of the collective modes of interfacial water, classical MD simulations were performed at Au(111)-water interfaces using the TIP4P/2005 model^39^ after equilibration at 300 K. To illustrate the inhomogeneous nature of the electrochemical interface, the polarizable Lennard-Jones potential was adopted for the Au atoms.^40^ As shown in ESI,† this simulation can reproduce the well-accepted orientational properties of interfacial waters^12,13,51–53^ with the appropriate interfacial energy. Briefly, when the surface is negatively charged, the water molecules are in the “H-down” configuration in the first layer; one H atom of water interacts with the Au surface rather than neighbouring water molecules. In contrast, when the surface is positively charged, the water molecules are in the “H-up” configuration in the first layer, meaning that two H atoms of the “H-up” water participate in the bulk network. Based on this validation, the THz responses of these interfacial water molecules were evaluated using the power spectral densities (PSDs) for the first-layer of water molecules on Au(111) with different surface charges. The computed PSDs, namely the vibrational densities of states, should provide a good approximation of the experimentally obtained vibrational SERS susceptibility spectra in the THz region, because low-frequency Raman scattering of water dominantly measures fluctuated changes in the distance between oxygen atoms of neighbouring water molecules. Fig. 3a shows the differential PSDs of the total velocities of interfacial water for the negatively and positively charged surfaces with respect to the neutral surface (pzc). In-plane motions and out-of-plane motions of the interfacial water molecules are also presented in Fig. 3b and c, respectively. The overall trend in the simulated spectral changes indeed resembles the potential induced changes observed in the Δ*χ*′′_SERS_ spectra (note that Fig. 3 does not include the contribution of electronic Raman responses in SERS. See also Fig. S10†). For example, the increase of the *ν*53 peak and the fast Debye relaxation towards negative potentials in Fig. 2b is found similarly in the negative ΔPSD, especially for the in-plane motion in Fig. 3b. The increase of the T + R mode at around 200 cm^−1^ under the positive-going scan is also seen in the positive ΔPSD, especially for the out-of-plane motion in Fig. 3c. For more microscopic interpretations, the distribution function of the total lifetime of hydrogen-bonds in a configuration, *P*_TC_(*t*),^24^ was calculated using the MD simulation results. As shown in Fig. 3d–f, the probability distribution of the hydrogen-bond lifetime increased at around 1 ps on the negatively charged surface. Such breaking of hydrogen-bonds creates a defect in the hydrogen-bond network, leading to local fluctuations of the network. This local fluctuation causes the change of polarizability of water that activates the Raman components in the THz region.^25^ This is indeed very convincing that the increased population of “H-down” water molecules on the negatively charge surface can induce hydrogen-deficient local defects within the first-layer water network. On the other hand, the increased population of “H-up” water molecules on the positively charged surface will result in the enhancement of the out-of-plane motion through the participation of two H atoms of water in the network, *i.e.*, formation of hydrogen-excess local defects.

**Fig. 4:**
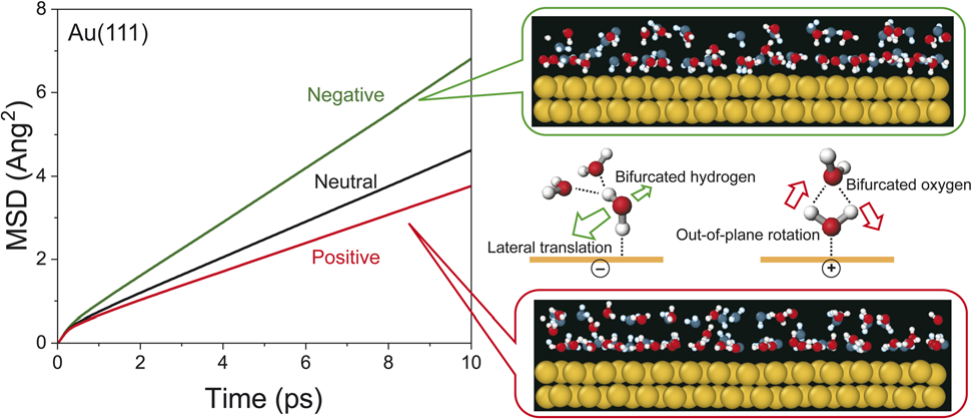
The MSD plot of water molecules within 0.3 nm from the surfaces of negatively charged, neutral, and positively charged Au(111). The side-view snapshots (two atomic rows depth of Au) are overlaid with the time-ordered sequence of positions of water molecules at 0 ps (blue) and 1 ps (red) on the negatively and positively charged Au(111).

The potential-induced change in the lifetime of hydrogen-bonds, suggested by both SERS susceptibility spectra and MD simulations, can affect the mobility of water molecules on the surface. This is indeed confirmed by the mean squared displacement (MSD) plots for the interfacial water molecules, obtained from the simulation results. As shown in Fig. 4, the lateral mobility of interfacial water molecules is much larger in the “H-down” configuration than in the “H-up” configuration. Such asymmetric behaviour with respect to the applied potential has also been reported in *ab initio* MD simulations.^54^ Apparently, the disengagement of one H atom in the “H-down” water molecules from the hydrogen-bond networks, *i.e.*, the generation of so-called bifurcated hydrogen defects (hydrogen-deficient defects),^25^ leads to an increase in the translational vibration of the first-layer water molecules along the surface. In other words, defect migration is enhanced on the negatively charged surface; this is consistent with the increased response of the fast Debye relaxation and the in-plane motions of the T mode on the negatively charged surface. Conversely, the participation of two H atoms of the “H-up” water molecules in the upper-layer hydrogen bonded networks creates bifurcated oxygen defects (hydrogen-excess defects)^25^ in the network, resulting in an increase of out-of-plane rotational vibration of the first-layer water molecules, as indicated by the decrease in the lateral mobility and the increase in the out-of-plane motions of the T + R mode on the positively charge surface.

## Conclusions

The dynamic behaviour of interfacial water molecules was studied using the newly developed surface-selective THz vibrational spectroscopy and MD simulations. The inhomogeneous nature of water molecules at electrified interfaces was clearly observed as the potential-dependent changes in the lifetime of the hydrogen-bonds of interfacial water molecules. When the applied potential was more negative than the pzc, the lateral motions of the “H-down” water molecules were accelerated with the increased bifurcated hydrogen defects in the local hydrogen-bond network. When the applied potential was more positive than the pzc, the collective motions of the “H-up” water molecules were more intensified along the out-of-plane direction due to the increased bifurcated oxygen defects in the network. Given that the Marcus theory demonstrates enormous effects of the reorganization energy of solvent molecules on electron transfer rates, such interface-specific dynamic behaviour of water should affect the kinetic behaviour of various electrochemical reactions such as hydrogen evolution and oxygen evolution reactions. We believe that the direct measure of the interfacial THz responses will shed new light on fundamental and applied studies of aqueous electrochemical and biological systems.

## Data availability

All additional data are in the ESI† associated with this publication.

## Author contributions

K. Ikeda conceived the project, T. Isogai performed SERS studies, M. Uranagase and S. Ogata performed MD simulations, K. Motobayashi performed electrochemical studies, K. Ikeda performed data analysis and drafted the manuscript, and all co-authors contributed to reviewing and editing the manuscript into its final format.

## Conflicts of interest

There are no conflicts to declare.

## Supplementary Material

SC-014-D3SC01734F-s001
