# Supplementary material for: Probing collective terahertz vibrations of a hydrogen-bonded water network at buried electrochemical interfaces
Source: Chem Sci. 2023 May 15;14(24):6531–7. doi: 10.1039/d3sc01734f (PMC10284101; doi:10.1039/d3sc01734f)
Supplement: SC-014-D3SC01734F-s001 [file SC-014-D3SC01734F-s001.pdf]

Electronic Supplementary Material (ESI) for Chemical Science.
This journal is © The Royal Society of Chemistry 2023

Supplementary Information:

Probing collective terahertz vibrations of hydrogen-bonded water network at buried electrochemical interfaces

Taichi Isogai,[1] Masayuki Uranagase,[1] Kenta Motobayashi,[1] Shuji Ogata,[1] and Katsuyoshi Ikeda[*,1,2]

[1]Department of Physical Science and Engineering, Nagoya Institute of Technology, Gokiso, Showa, Nagoya 466-8555, Japan. [2]Frontier Research Institute for Materials Science (FRIMS), Nagoya Institute of Technology, Gokiso, Showa, Nagoya 466-8555, Japan.

*e-mail: kikeda@nitech.ac.jp

1. Extraction of Raman susceptibilities from normal Raman and SERS spectra[32-34]

The absolute wavenumber ($\tilde{\nu} > 0$ in cm^{-1}) and the relative wavenumber ($\tilde{\nu}^{\Delta}$ in cm^{-1}) are used in the following description by considering the connection to traditional wavenumber plotting. A power spectrum for normal electronic Raman scattering (ERS), $I_{\mathrm{ERS}}(\tilde{\nu}^{\Delta})$, is connected with the imaginary part of the dynamic susceptibility, $\chi''_{\mathrm{ERS}}(\tilde{\nu})$, by the following expressions:

$$I_{\mathrm{ERS}}(\tilde{\nu}_{\mathrm{as}}^{\Delta}) = K \cdot (\tilde{\nu}_0 + \tilde{\nu})^3 \cdot [n(\tilde{\nu})] \cdot \chi''_{\mathrm{ERS}}(\tilde{\nu}) \tag{1}$$

for anti-Stokes branch and

$$I_{\mathrm{ERS}}(\tilde{\nu}_{\mathrm{s}}^{\Delta}) = K \cdot (\tilde{\nu}_0 - \tilde{\nu})^3 \cdot [n(\tilde{\nu}) + 1] \cdot \chi''_{\mathrm{ERS}}(\tilde{\nu}) \tag{2}$$

for Stokes branch of the spectrum. Here, $\tilde{\nu}_0$ is the photon energy for the incident laser ($\tilde{\nu}_0$ = 1/632.8 nm = 15802.8 cm^{-1} (1.96 eV) in the present case). $\tilde{\nu}$ is the energy exchanged between photon and electron, *i.e.*, the energy difference between the initial and final states of scattered electrons in the conduction band of metal. $\tilde{\nu}_{\mathrm{as}}^{\Delta}$ and $\tilde{\nu}_{\mathrm{s}}^{\Delta}$ are the Raman shifts relative to $\tilde{\nu}_{\mathrm{i}}$ in the anti-Stokes and Stokes branches, *i.e.*, $\tilde{\nu}_{\mathrm{as}}^{\Delta} = -\tilde{\nu}$ and $\tilde{\nu}_{\mathrm{s}}^{\Delta} = \tilde{\nu}$. K is an instrument function. $[n(\tilde{\nu})]$ and $[n(\tilde{\nu}) + 1]$ correspond to thermal factors for the anti-Stokes and Stokes processes, respectively, and $n(\tilde{\nu})$ is the Bose-Einstein distribution described as $n(\tilde{\nu}) = [\exp(hc\tilde{\nu}/k_{\mathrm{B}}T) - 1]^{-1}$, where h, c and k_{B} are Planck's constant, the

velocity of light and the Boltzmann constant, respectively. A power spectrum for normal vibrational Raman scattering (VRS), $I_{\mathrm{VRS}}(\tilde{\nu}^{\Delta})$, is connected with the imaginary part of the dynamic susceptibility, $\chi''_{\mathrm{VRS}}(\tilde{\nu})$, in a similar manner:

$$I_{\mathrm{VRS}}(\tilde{\nu}_{\mathrm{as}}^{\Delta}) = K \cdot (\tilde{\nu}_0 + \tilde{\nu})^3 \cdot [n(\tilde{\nu}) \quad\quad] \cdot \chi''_{\mathrm{VRS}}(\tilde{\nu}) \tag{3}$$

for anti-Stokes branch and

$$I_{\mathrm{VRS}}(\tilde{\nu}_{\mathrm{s}}^{\Delta}) = K \cdot (\tilde{\nu}_0 - \tilde{\nu})^3 \cdot [n(\tilde{\nu}) + 1] \cdot \chi''_{\mathrm{VRS}}(\tilde{\nu}) \tag{4}$$

for Stokes branch of the spectrum.

For SERS, plasmonic enhancement of ERS, *i.e.*, ESERS, is described as:

$$I_{\mathrm{ESERS}}(\tilde{\nu}_{\mathrm{as}}^{\Delta}) = K \cdot (\tilde{\nu}_0 + \tilde{\nu})^3 \cdot [n(\tilde{\nu}) \quad\quad] \cdot \left[\frac{E_{\mathrm{loc}}^{\mathrm{m}}(\tilde{\nu}_0+\tilde{\nu})}{E_0(\tilde{\nu}_0+\tilde{\nu})}\right]^2 \cdot \left[\frac{E_{\mathrm{loc}}^{\mathrm{m}}(\tilde{\nu}_0)}{E_0(\tilde{\nu}_0)}\right]^2 \cdot g^{\mathrm{m}} \cdot \chi''_{\mathrm{ERS}}(\tilde{\nu}) \tag{5}$$

for anti-Stokes branch and

$$I_{\mathrm{ESERS}}(\tilde{\nu}_{\mathrm{s}}^{\Delta}) = K \cdot (\tilde{\nu}_0 - \tilde{\nu})^3 \cdot [n(\tilde{\nu}) + 1] \cdot \left[\frac{E_{\mathrm{loc}}^{\mathrm{m}}(\tilde{\nu}_0-\tilde{\nu})}{E_0(\tilde{\nu}_0-\tilde{\nu})}\right]^2 \cdot \left[\frac{E_{\mathrm{loc}}^{\mathrm{m}}(\tilde{\nu}_0)}{E_0(\tilde{\nu}_0)}\right]^2 \cdot g^{\mathrm{m}} \cdot \chi''_{\mathrm{ERS}}(\tilde{\nu}) \tag{6}$$

for Stokes branch. Similarly, plasmonic enhancement of VRS, *i.e.*, VSERS, is described as:

$$I_{\mathrm{VSERS}}(\tilde{\nu}_{\mathrm{as}}^{\Delta}) = K \cdot (\tilde{\nu}_0 + \tilde{\nu})^3 \cdot [n(\tilde{\nu}) \quad\quad] \cdot \left[\frac{E_{\mathrm{loc}}^{\mathrm{d}}(\tilde{\nu}_0+\tilde{\nu})}{E_0(\tilde{\nu}_0+\tilde{\nu})}\right]^2 \cdot \left[\frac{E_{\mathrm{loc}}^{\mathrm{d}}(\tilde{\nu}_0)}{E_0(\tilde{\nu}_0)}\right]^2 \cdot g^{\mathrm{d}} \cdot \chi''_{\mathrm{VRS}}(\tilde{\nu}) \tag{7}$$

for anti-Stokes branch and

$$I_{\mathrm{VSERS}}(\tilde{\nu}_{\mathrm{s}}^{\Delta}) = K \cdot (\tilde{\nu}_0 - \tilde{\nu})^3 \cdot [n(\tilde{\nu}) + 1] \cdot \left[\frac{E_{\mathrm{loc}}^{\mathrm{d}}(\tilde{\nu}_0-\tilde{\nu})}{E_0(\tilde{\nu}_0-\tilde{\nu})}\right]^2 \cdot \left[\frac{E_{\mathrm{loc}}^{\mathrm{d}}(\tilde{\nu}_0)}{E_0(\tilde{\nu}_0)}\right]^2 \cdot g^{\mathrm{d}} \cdot \chi''_{\mathrm{VRS}}(\tilde{\nu}) \tag{8}$$

for Stokes branch. Here, g^{m} and g^{d} denote the coupling efficiencies of Raman transitions to the plasmonic cavity in the metal phase and dielectric phase, respectively; g^{d} explains the surface selection rules because the coupling efficiency is affected by the orientation and the position of molecular dipoles in the cavity. On the other hand, g^{m} would be less sensitive to $\tilde{\nu}_{\mathrm{m}}$ because free electrons are the source of the signals. For ESERS, therefore, the experimentally obtained $I_{\mathrm{ESERS}}/I_{\mathrm{ERS}}$, corresponds to the radiative Purcell factor as follows:

$$I_{\mathrm{ESERS}}/I_{\mathrm{ERS}} = \left[\frac{\mathrm{E}_{\mathrm{loc}}^{\mathrm{m}}(\tilde{\nu}_0+\tilde{\nu})}{\mathrm{E}_0(\tilde{\nu}_0+\tilde{\nu})}\right]^2 \cdot \left[\frac{\mathrm{E}_{\mathrm{loc}}^{\mathrm{m}}(\tilde{\nu}_0)}{\mathrm{E}_0(\tilde{\nu}_0)}\right]^2 \cdot \mathrm{g}^{\mathrm{m}} \propto \left[\frac{\mathrm{E}_{\mathrm{loc}}^{\mathrm{m}}(\tilde{\nu}_0+\tilde{\nu})}{\mathrm{E}_0(\tilde{\nu}_0+\tilde{\nu})}\right]^2. \tag{9}$$

Now, when the Raman frequency shift $\tilde{\nu}$ is small, one can expect the ratio of $\mathrm{EF}^{\mathrm{VSERS}}$ to $\mathrm{EF}^{\mathrm{ESERS}}$ is nearly constant: $\mathrm{EF}^{\mathrm{VSERS}}/\mathrm{EF}^{\mathrm{ESERS}} \sim \mathrm{C}$. Then, we obtain:

$$\begin{aligned} I_{\mathrm{SERS}}(\tilde{\nu}_{\mathrm{as}}^{\Delta}) &= I_{\mathrm{ESERS}}(\tilde{\nu}_{\mathrm{as}}^{\Delta}) + I_{\mathrm{VSERS}}(\tilde{\nu}_{\mathrm{as}}^{\Delta}) \\ &= K \cdot (\tilde{\nu}_0 + \tilde{\nu})^3 \cdot [n(\tilde{\nu}) \quad\quad] \cdot I_{\mathrm{ESERS}}/I_{\mathrm{ERS}} \cdot (\chi''_{\mathrm{ERS}}(\tilde{\nu}) + C \cdot g^{\mathrm{d}} \cdot \chi''_{\mathrm{VRS}}(\tilde{\nu})) \\ &= K \cdot (\tilde{\nu}_0 + \tilde{\nu})^3 \cdot [n(\tilde{\nu}) \quad\quad] \cdot I_{\mathrm{ESERS}}/I_{\mathrm{ERS}} \cdot \chi''_{\mathrm{SERS}}(\tilde{\nu}) \end{aligned} \tag{10}$$

for anti-Stokes branch and

$$I_{\mathrm{SERS}}(\tilde{\nu}_{\mathrm{s}}^{\Delta}) = I_{\mathrm{ESERS}}(\tilde{\nu}_{\mathrm{s}}^{\Delta}) + I_{\mathrm{VSERS}}(\tilde{\nu}_{\mathrm{s}}^{\Delta})$$

$$= K \cdot (\tilde{\nu}_0 - \tilde{\nu})^3 \cdot [n(\tilde{\nu}) + 1] \cdot I_{\mathrm{ESERS}}/I_{\mathrm{ERS}} \cdot (\chi''_{\mathrm{ERS}}(\tilde{\nu}) + C \cdot g^{\mathrm{d}} \cdot \chi''_{\mathrm{VRS}}(\tilde{\nu}))$$

$$= K \cdot (\tilde{\nu}_0 - \tilde{\nu})^3 \cdot [n(\tilde{\nu}) + 1] \cdot I_{\mathrm{ESERS}}/I_{\mathrm{ERS}} \cdot \chi''_{\mathrm{SERS}}(\tilde{\nu}) \quad (11)$$

for Stokes branch, where $\chi''_{\mathrm{SERS}}(\tilde{\nu}) \equiv \chi''_{\mathrm{ERS}}(\tilde{\nu}) + C \cdot g^{\mathrm{d}} \cdot \chi''_{\mathrm{VRS}}(\tilde{\nu})$.

2. Electrochemical and spectroscopic measurements

The cleanness of the electrochemical cell was confirmed by measuring cyclic voltammograms (CVs) of Au(111) surfaces in sulfuric acid solution, because the characteristic electrochemical responses of Au are highly sensitive to the presence of impurities. Although the Ag/AgCl reference electrode was used in this study, the possibility of the specific adsorption of Cl^- on Au surface can be excluded by disappearance of νAu-Cl^- at 265 cm^{-1} in the SERS spectra after the surface cleaning process.

The values of pzc for rough Au surfaces in the different electrolyte solutions were experimentally determined using the capacity curve measurements in the double layer region,[29] as shown in Fig. S1.

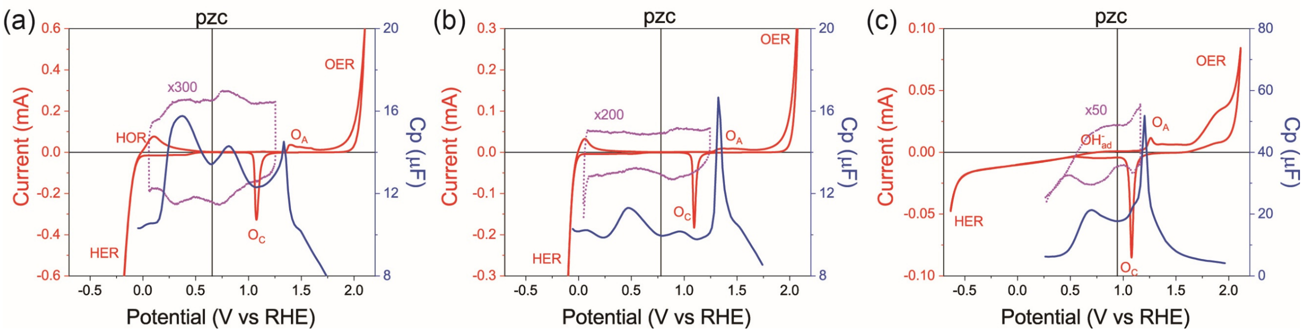

Fig. S1. Cyclic voltammograms (CVs) in the double layer region (purple) and wide potential region (red), and capacity curves (blue) measured on SERS-active rough Au surfaces in different electrolyte solutions. OER: oxygen evolution reaction, HER: hydrogen evolution reaction, HOR: hydrogen oxidation reaction, O_A: anodic formation of Au oxide, O_C: cathodic reduction of Au oxide. The CVs were measured with the scan rate of 50 mV/s. The capacity curves were taken at 10 Hz with 10 mV modulation for the positive-going potential steps. **(a)** 0.1 M H_2SO_4 aqueous solution. **(b)** 0.2 M $HClO_4$ aqueous solution. **(c)** 0.1 M KOH aqueous solution.

The individual responses for ERS from an Au substrate and VRS from an electrolyte solution are presented in Fig. S2a. The electronic Raman susceptibility (χ''_{ERS}) spectrum for the SERS-inactive smooth Au surface and the vibrational Raman susceptibility (χ''_{VRS}) spectrum for a 0.2 M $HClO_4$ aqueous bulk solution were obtained by reducing the Bose-Einstein thermal factor in the spontaneous Raman spectra (Eqs. 1-4). The symmetry of the responses between the Stokes and anti-Stokes branches, which is required by the time-reversal symmetry between these Raman processes,[45] was confirmed for both spectra. In the χ''_{ERS} spectrum for Au, a broad feature with a maximum at around 670 cm^{-1} (20 THz) was found as in a previous report.[32,34] In the χ''_{VRS} spectrum for the acidic solution, several vibrational features are observed. For vibrations of the electrolyte species, the sharp peaks at 1102, 934, 625, and 462 cm^{-1} are assigned to antisymmetric stretching (F2), symmetric stretching (A1), antisymmetric bending (F2), and symmetric bending (E) of the perchlorate anion with T_d symmetry. For the water vibrations, both the high-frequency local modes and the low-frequency collective modes are clearly observed; for example, in the fingerprint region, H-O-H bending is seen at 1640 cm^{-1}. Libration modes are also observed as a very broad feature in the range 400 - 800 cm^{-1}. In the THz-shifted region, two broad bands are observed at 48 cm^{-1} (1.4 THz) and 160 cm^{-1} (4.8 THz), which are denoted as T and T+R.

The surface-enhanced Raman responses at the electrode-electrolyte interface and their dependence on the electrochemical potential are presented in Fig. S2b, which can be obtained by reducing the Purcell factor as well as the Bose-Einstein thermal factor (Eqs. 10, 11). The trend in the spectral features of χ''_{SERS} would appear to be, roughly speaking, the sum of χ''_{ERS} for Au and χ''_{VRS} for the aqueous solution. The background intensity, i.e., χ''_{ERS} for Au, is substantially decreased when the applied potential is more positive than the pzc; this is well explained by the potential-induced change of the surface excess charge of Au.[29,46-48] In the high-frequency fingerprint region of χ''_{SERS}, several vibrational features are observed: the symmetric stretch of perchlorate anion, $\nu_S(ClO_4^-)$, is probably

due to the contribution from the bulk solution because the peak intensity is nearly invariant during the potential scan. The peaks at 1190 and 1370 cm^{-1} are ascribed to the adsorbed perchlorate ions.

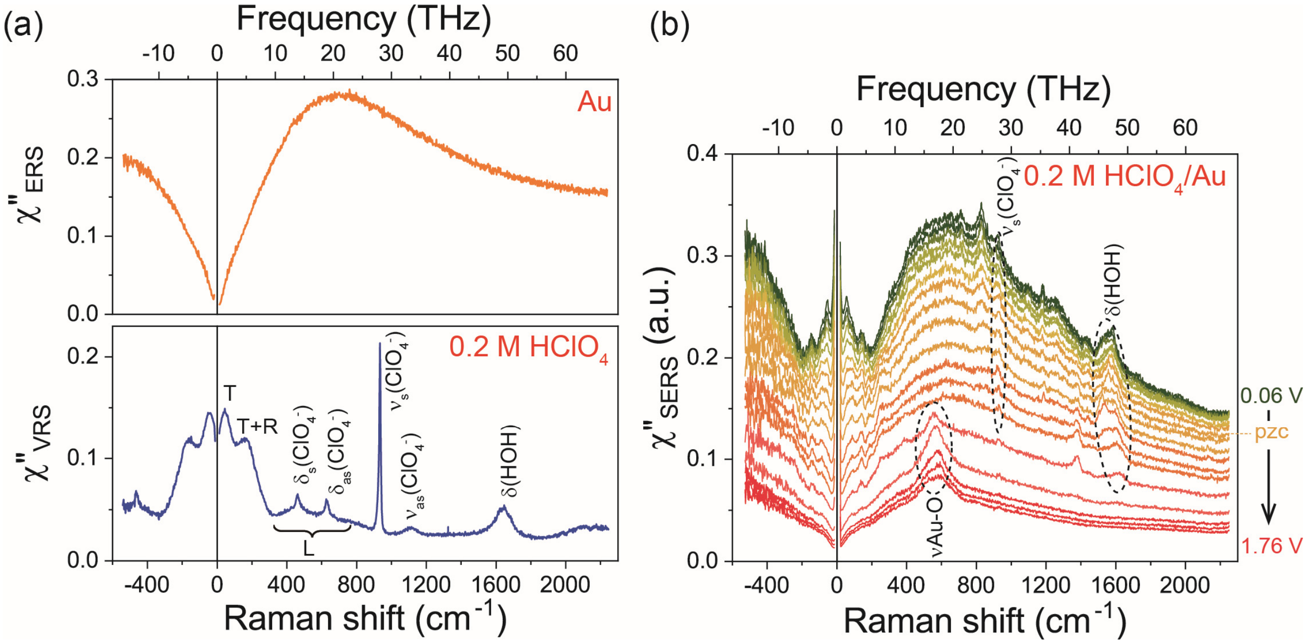

Fig. S2. Raman susceptibilities for electrode, electrolyte, and their interface. (a) Electronic Raman susceptibility (χ''_{ERS}) spectrum for smooth Au surface (upper panel) and vibrational Raman susceptibility (χ''_{VRS}) spectrum for 0.2M $HClO_4$ aqueous solution (bottom panel). L: librations of water, T: collective translational mode, T+R: collective translational and rotational mode. **(b)** A series of surface-enhanced Raman susceptibility (χ''_{SERS}) spectra for 0.2 M $HClO_4$ aqueous solution at a SERS-active Au surface obtained under positive-going potential scan (pzc = 0.76 V vs. RHE).

Fig. S3 shows the potential dependence of χ''_{SERS} and $\Delta\chi''_{SERS}$ spectra obtained for Au surface in various electrolyte solutions. The Stokes and anti-Stokes branches in the spectra are symmetric for both vibrational features and background continuum, indicating that the densities of states formats for Raman scattering are properly obtained by reducing the temperature, frequency, and Purcell factors. The THz-shifted Stokes branches in these spectra are enlarged in Fig. 2.

To confirm that these THz vibrational features are indeed originated from interfacial water molecules, χ''_{SERS} spectrum at Au/gas interfaces was compared under different humidity. As shown in Fig. S4, the THz-vibrational features reversibly decreased and increased in intensity under flow of

dry Ar gas (purity of 99.999%) and of humid air (RH40%), respectively. On the other hand, the peak intensity of νAu-Cl$^-$, which was originated from residual chloride adsorbates, was not influenced by humidity, indicating that SERS effect remains intact during the experiments.

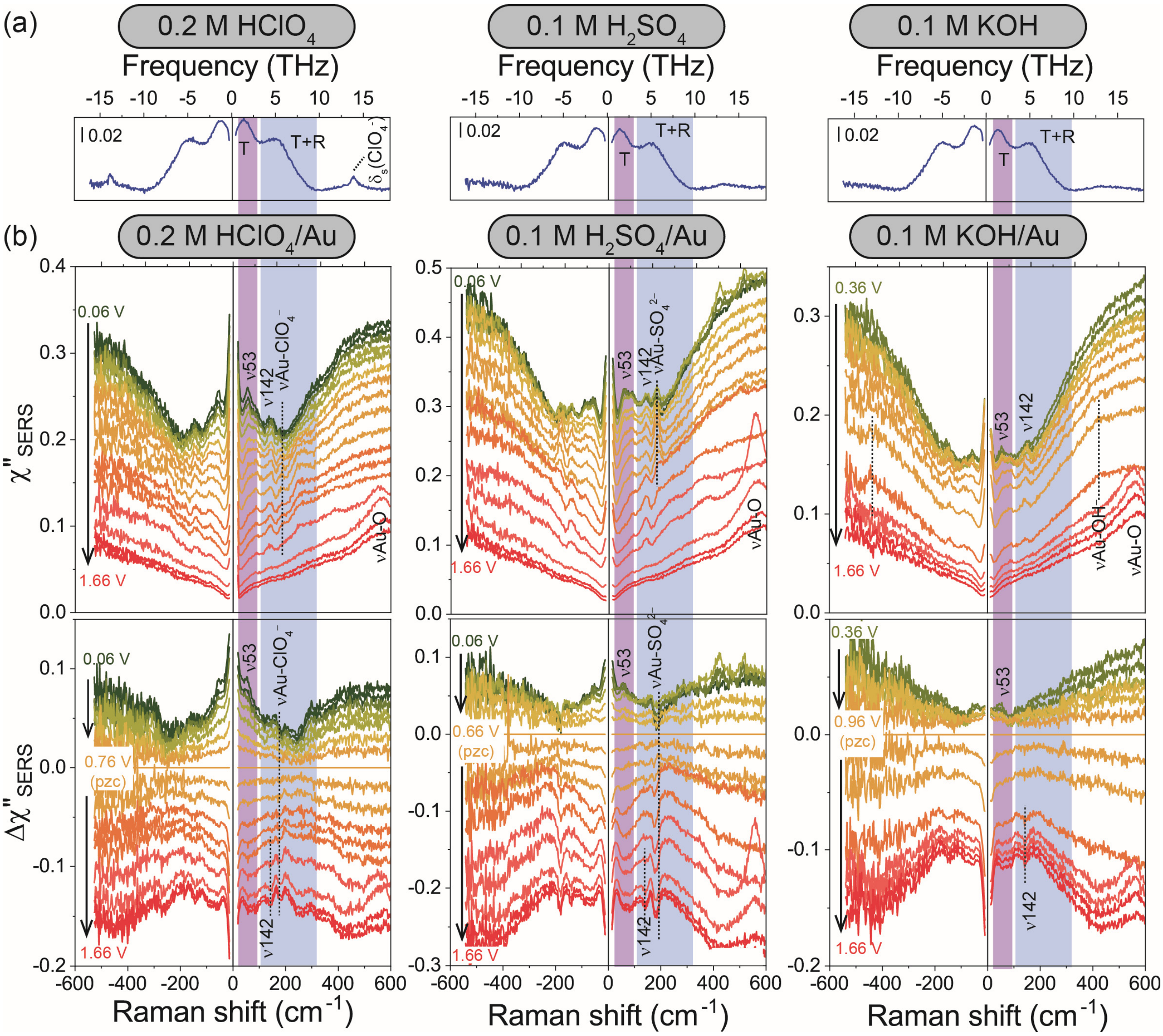

Fig. S3. Potential dependence of surface-enhanced Raman susceptibility (χ"$_{SERS}$) spectra in the THz-shifted region (upper panel) and their difference (Δχ"$_{SERS}$) spectra (bottom). (a) χ"$_{VRS}$ spectra for bulk aqueous solutions containing 0.2M $HClO_4$, 0.1M H_2SO_4, and 0.1M KOH. **(b)** Potential dependence of χ"$_{SERS}$ spectra for the respective electrolyte solutions on Au surface and their differential spectra with respect to the pzc for each electrolyte solution.

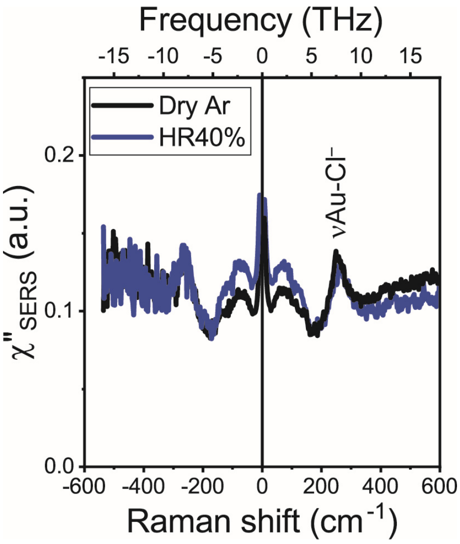

Fig. S4. Humidity dependence of surface-enhanced Raman susceptibility (χ''_{SERS}) spectra for Au/gas interface in the THz-shifted region.

3. Simulation method

In all of the MD simulations presented, water molecules were treated using the TIP4P/2005 model,[38] which represented each water molecule as a rigid body. As shown in Fig. S5, this model well reproduced the collective behaviour of water molecules in the THz frequency region. According to the results of the MD simulation, the T mode is assigned to a collective translational mode of water molecules. This mode can be interpreted as the intermolecular bending mode because a whole water molecule moves relative to the other water molecules. The T+R mode is ascribed to the collective mode of water molecules featuring a combination of translational and rotational motions, which corresponds to the stretching mode of intermolecular O-O.

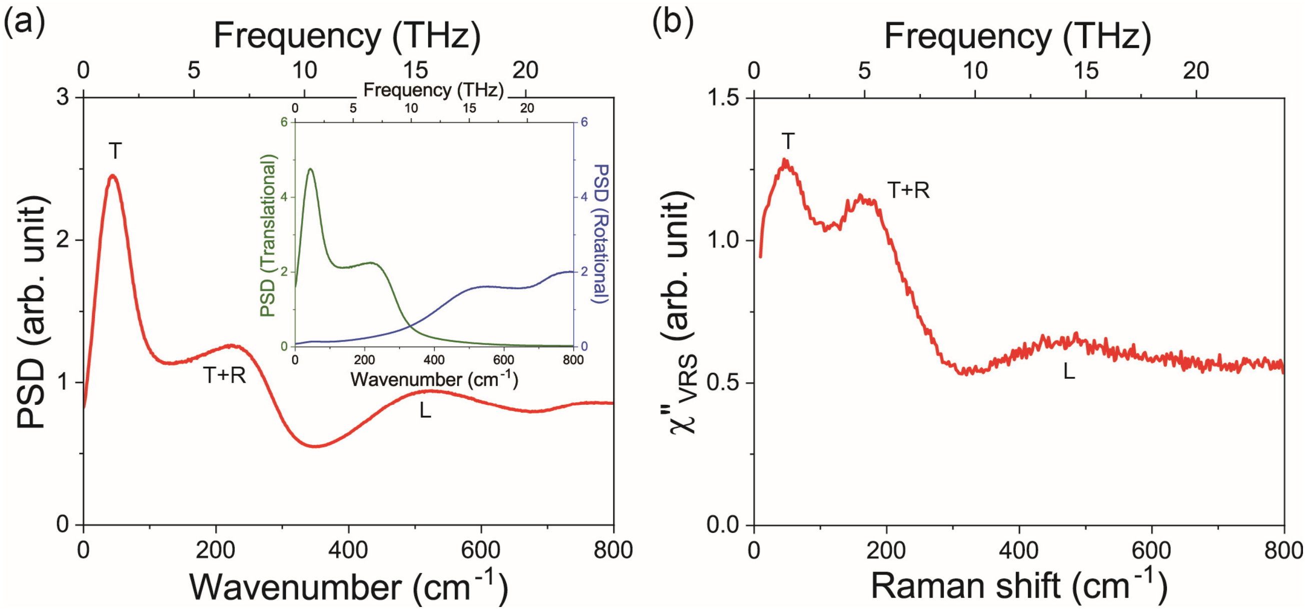

Fig. S5. Vibrational spectra of bulk water. **(a)** Simulated PSD of bulk water. Inset: translational and rotational PSD of bulk water. **(b)** Experimentally obtained vibrational Raman susceptibility (χ''_{VRS}) spectrum of bulk water. T: collective translational mode, T+R: collective translational and rotational mode, L: librations of water.

For simulation of the electrochemical interfaces, 4000 water molecules were inserted between two Au slabs located parallel to each other at a distance of 7.6 nm (Fig. S6). The z axis is chosen as the direction perpendicular to the interface between the Au and the water. Thus, for the Au(111) surface, the x, y, and z axes are defined by the direction parallel to [-110], [11-2], and [111], respectively. The size of each slab is 4.04 nm, 4.00 nm, and 1.88 nm in the x, y, and z directions, respectively. For Au(100), the x, y, and z axes are defined by the direction parallel to [010], [001], and [100], respectively. The size of each slab is 4.08 nm, 4.08 nm, and 1.63 nm in the x, y, and z directions, respectively. Periodic boundary conditions are applied for all directions. The dimension of the simulation cell in z direction is set to 20 nm, while those in the x and y directions are determined in order to accommodate the gold slabs.

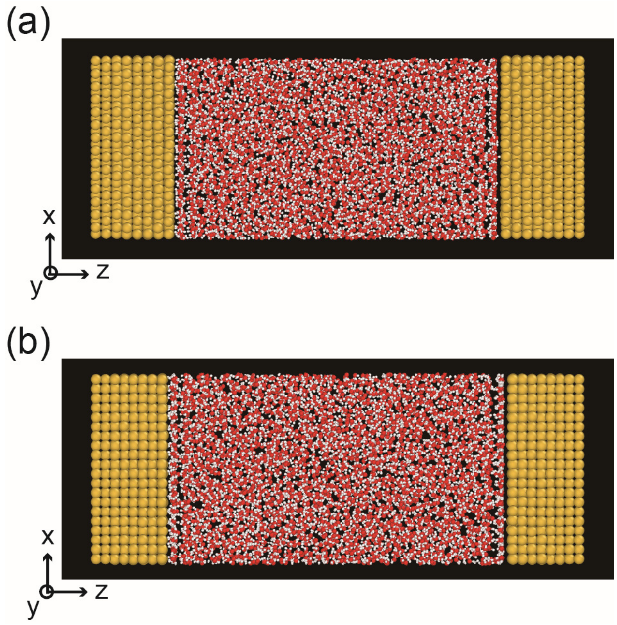

Fig. S6. Simulation cells with two Au slabs and 4000 water molecules for simulating the electrochemical interfaces. **(a)** Au(111). **(b)** Au(100).

To illustrate the inhomogeneous nature of the electrochemical interface, the polarizable Lennard-Jones potential reported by Geada *et al.*[40] was adopted for the Au atoms. In this model, each atom consists of a core and a dummy electron, which are connected through a harmonic spring. A charged Au surface is prepared by adjusting the charge of the cores of the Au atoms on the surface. When one Au slab is positively charged, the other slab is negatively charged so that the entire system maintains charge neutrality. The short-range interactions between water and Au atoms are treated using the Lennard-Jones potential with the parameters obtained according to the Lorentz-Berthelot rule. The cut-off length of the Lennard-Jones potential was set to five times of the diameter appeared in expression of the potential. The Coulomb potential was evaluated by the standard Ewald method. (The incorporation of the ions in the simulation was practically difficult because the concentration of 0.1 M corresponds to only seven ions in 4000 water molecules, which leads large fluctuation of the calculation results. However, the applied charge of 0.11 C/m^2 on the Au surface corresponds to the

electric field of 150 MV/m, which is comparable to the electric field expected in the double layer under potential application of 1V with respect to pzc.)

The system was first equilibrated for 1 ns under a canonical ensemble by maintaining the temperature at 300 K using the velocity rescaling method developed by Bussi *et al.*[41] Subsequently, the simulation for data production was conducted for 1 ns under a microcanonical ensemble; data were collected every 20 fs. The equations of motion were integrated numerically using the velocity Verlet method with a time step of 2.5 fs. The Au atoms in the two layers from the surface which were not in contact with water were fixed. The configuration of the dummy electrons on the surface Au was determined at every step by searching for the minimum energy state under the condition where all atoms were fixed using the conjugate gradient method.

The use of the TIP4P/2005 model in our simulations, which is different from the flexible SCP model by Geada et al,[40] was validated by calculating the gold-water interfacial energy at 300 K. In this calculation, a three-box approach was applied:[40] the first system is bulk water, the second is bulk gold, and the third is interfacial model by combining first two models. The number of water molecules and the surface size of the gold were set to the same value for the system displayed in Fig. S6, while the thickness of the gold was set to about 3 nm. The gold-water interfacial energy was obtained by subtracting the sum of the energies of the first two models from that for the third model. In our simulations, the gold-water interfacial energy was evaluated as 1149 mJ/m^3 for Au(111) and 1138 mJ/m^3 for Au(100), which were consistent with those reported by Geada *et al.*

From the collected data, the velocity autocorrelation function defined by

$$C_{\rm tot}^{\alpha}(t;z_-,z_+) = \sum_{i=1}^{3N_w} \langle m_i v_i^{\alpha}(t) v_i^{\alpha}(0) \Theta(z_i(0)-z_-)\Theta\big(z_+ - z_i(0)\big)\rangle$$

is calculated, where N_w is the number of water molecules, m_i, $z_i(t)$, and $v_i^{\alpha}(t)$ are the mass, the position in the z direction, and the velocity in the α direction at time t for atom i, respectively; the

angle bracket signifies the average of the sampling ensemble, and $\Theta(x) = 1$ if $x \geq 0$, otherwise it is 0. The translational and rotational autocorrelation functions defined by

$$C^{\alpha}_{\text{trans}}(t; z_-, z_+) = \sum_{i=1}^{N_w} \langle v^{\alpha}_{i,\text{com}}(t) v^{\alpha}_{i,\text{com}}(0) \Theta(z_i(0) - z_-) \Theta\big(z_+ - z_i(0)\big) \rangle$$

$$C^{\alpha}_{\text{rot}}(t; z_-, z_+) = \sum_{i=1}^{N_w} \langle \omega^{\alpha}_i(t) \omega^{\alpha}_i(0) \Theta(z_i(0) - z_-) \Theta\big(z_+ - z_i(0)\big) \rangle$$

are also calculated, where $v^{\alpha}_{i,\text{com}}(t)$ and $\omega^{\alpha}_i(t)$ are the velocity of the center of mass and the angular velocity at time t for molecule i, respectively. The power spectral density (PSD) is evaluated using the Fourier transform of the autocorrelation function from the Wiener-Khinchin theorem. The spectra presented in this paper correspond to the Fourier transform of $C(t)/C(0)$, where z_- and z_+ are selected such that only molecules within 0.3 nm from the surface are considered. The PSD obtained using the rigid water model is considered as a good approximation of Raman spectra of water in THz region because fluctuated changes in polarizability of liquid water, which give low-frequency Raman scattering, are mainly caused by the change in the distance between oxygen atoms of neighbouring water molecules. The distribution function of persistence times of hydrogen-bonds, $P(t)$,[22] was also calculated from the MD simulation results according to the geometric hydrogen-bond definition by Martiniano et al.[23] This probability distribution $P(t)$, counting the number of pairs of molecules which had an un-interrupted hydrogen-bond during the time t, was further converted to the probability distribution of the total lifetime of hydrogen-bonds in a configuration, $P_{\text{TC}}(t)$ using $P_{\text{TC}}(t) = tP(t)\langle\tau\rangle$, where the mean hydrogen-bond lifetime $\langle\tau\rangle = \int_0^{\infty} tP(t)dt$.

Figs. S7 and S8 show spatial distribution and orientation of water molecules near Au surfaces with different charges, calculated for Au(111) and Au(100). The similar trends between these different surfaces indicate that the orientational properties of interfacial water molecules are dominantly determined by the surface charges (the electric field) rather than by the surface structures. Thus, the

simulation results are expected to be comparable with the behaviour of water molecules on SERS-active rough Au surfaces. The collective vibrational properties of interfacial water molecules are also presented for Au(111) in Fig. 3 and for Au(100) in Fig. S9. Actually, there are quantitative differences between the results for these two surfaces, but the trends are similar in both the spectral features and the hydrogen-bond lifetime. Fig. S10 compares the ΔPSD spectra with the experimental spectra, in which the baseline of each ΔPSD spectrum is adjusted to be fitted with the potential-induced change in the electronic Raman responses of Au in the $\Delta\chi''_{SERS}$ spectra. Again, the overall treads in the simulated results resemble with the experimental spectra. The increase in the in-plane mobility on the negatively charge surface is found on Au(100) as well as on Au(111), as shown in Fig. S11, indicating that bifurcated hydrogen defects generated around H-down water molecules promotes such lateral mobility through the translational vibrations. On the other hand, this mobility of interfacial water molecules is much larger on Au(111) than on Au(100) regardless of the surface charge. This is related to the different lifetimes of hydrogen bonds between Au(100) and Au(111); $\langle\tau\rangle$ = 0.72 ps, 0.61 ps, and 0.60 ps on Au(111) with surface charge of 0, −0.11, and +0.11 C/m^2, respectively, and 1.18 ps, 0.98 ps, and 0.96 ps on Au(100) with surface charge of 0, −0.11, and +0.11 C/m^2, respectively.

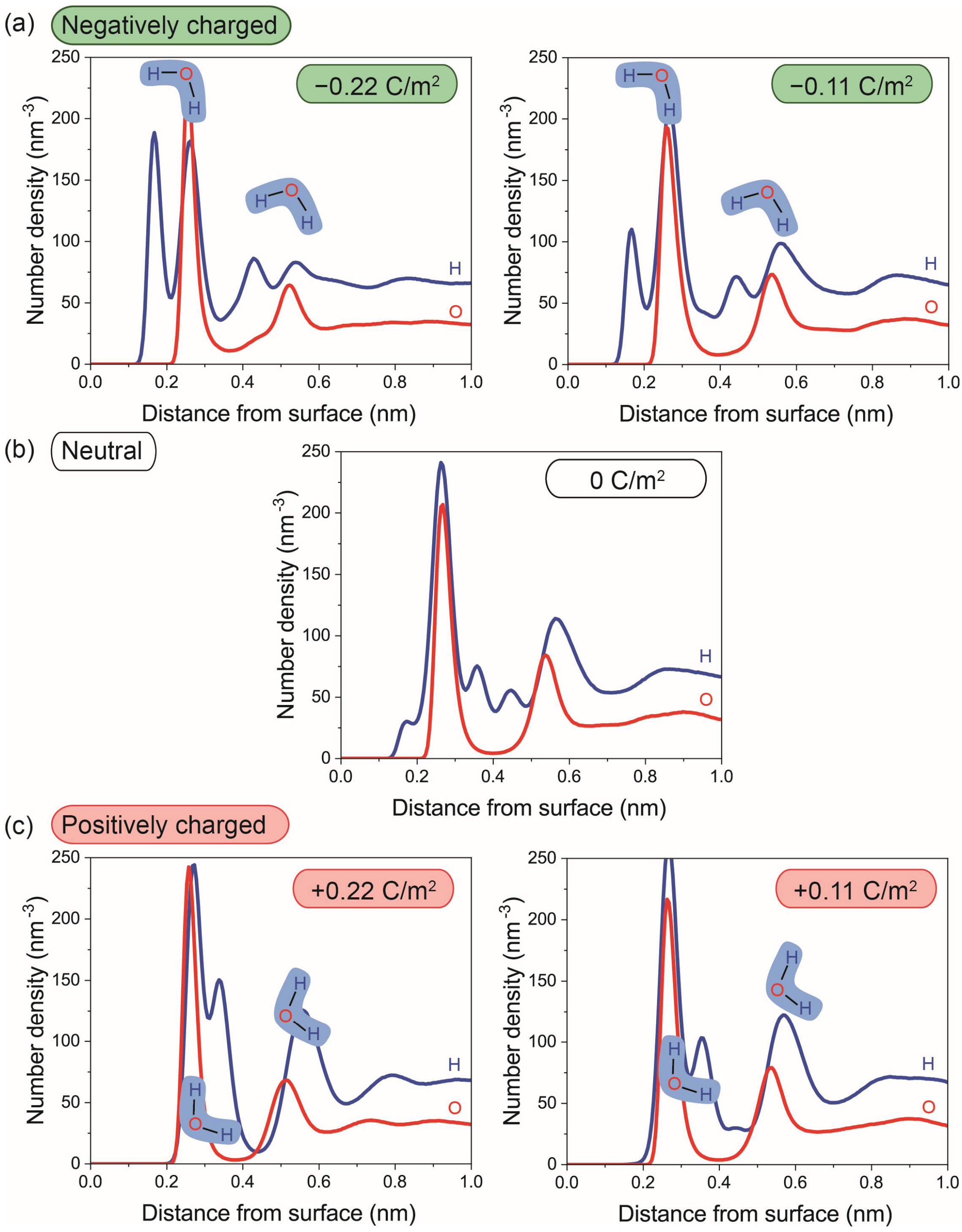

Fig. S7. Spatial distribution and orientation of water molecules near Au(111) surface with difference charges. **(a)** Number density of H and O atoms as a function of distance from negatively charged surfaces with the charge density of −0.22 and −0.11 C/m^2. **(b)** Number density distribution calculated on a neutral Au surface. **(c)** Number density distribution calculated on positively charged surface with the charge density of 0.22 and 0.11 C/m^2.

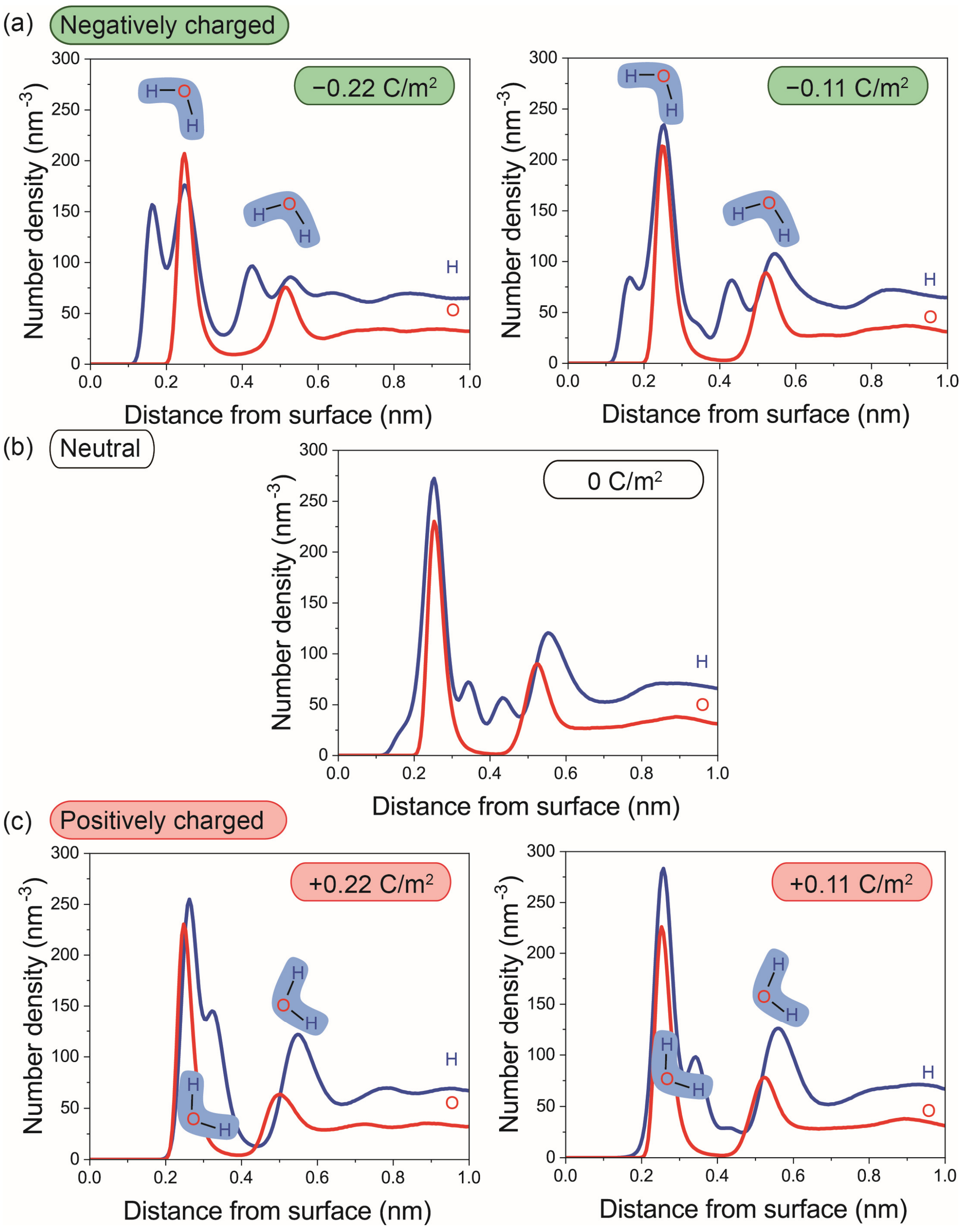

Fig. S8. Spatial distribution and orientation of water molecules near Au(100) surface with difference charges. **(a)** Number density of H and O atoms as a function of distance from negatively charged surfaces with the charge density of −0.22 and −0.11 C/m^2. **(b)** Number density distribution calculated on a neutral Au surface. **(c)** Number density distribution calculated on positively charged surface with the charge density of 0.22 and 0.11 C/m^2.

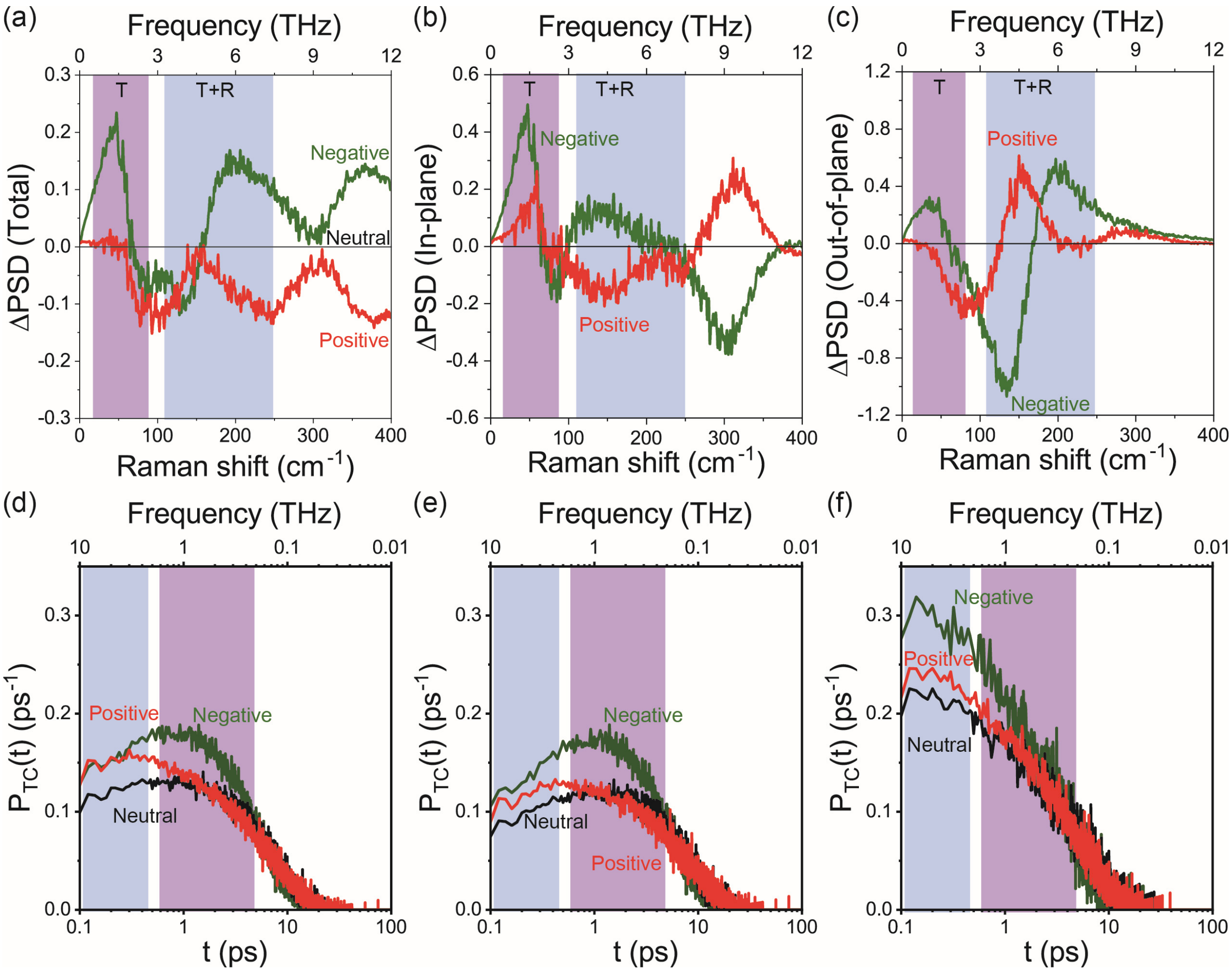

Fig. S9. Differential power spectral density (ΔPSD) for the first-layer water molecules on the Au(100) surface and probability distribution of total lifetime of hydrogen bonds (P_{TC}). (a) ΔPSD of total velocities of water molecules on negatively and positively charged surfaces with respect to the neutral surface. **(b)** In-plane motions of water network. **(c)** Out-of-plane motions of water network. **(d)** $P_{TC}(t)$ for total hydrogen-bonds of the first-layer water molecules. **(e)** $P_{TC}(t)$ for in-plane hydrogen-bonds of the first-layer water molecules. **(f)** $P_{TC}(t)$ for out-of-plane hydrogen-bonds of the first-layer water molecules. The surface charge of 0.11 C/m^2 was applied in the simulation.

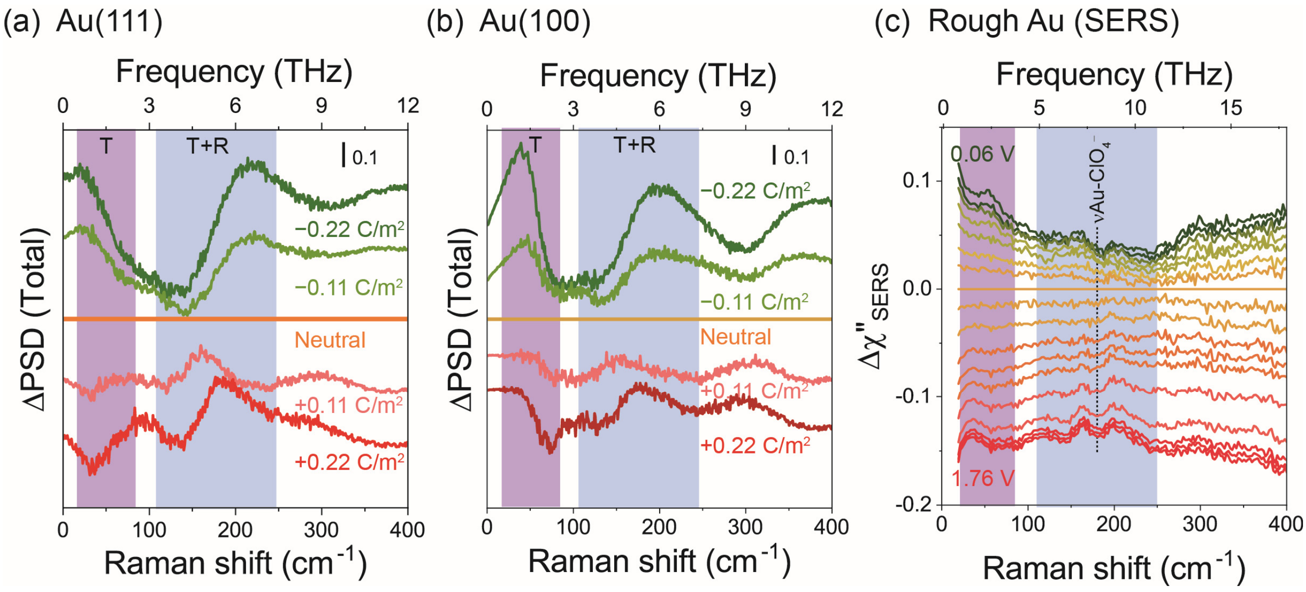

Fig. S10. Comparison between ΔPSD and Δχ”$_{SERS}$ spectra. (a) ΔPSD of total velocities of water molecules on differently charged surfaces of Au(111) with respect to the neutral surface with a constant baseline shift. **(b)** ΔPSD of total velocities of water molecules on differently charged surfaces of Au(100) with respect to the neutral surface with a constant baseline shift. **(c)** Δχ”$_{SERS}$ with respect to pzc, obtained from SERS spectra of rough Au in $HClO_4$ solution, in which the potential variation of the baseline is due to the change in the electronic Raman response of Au.

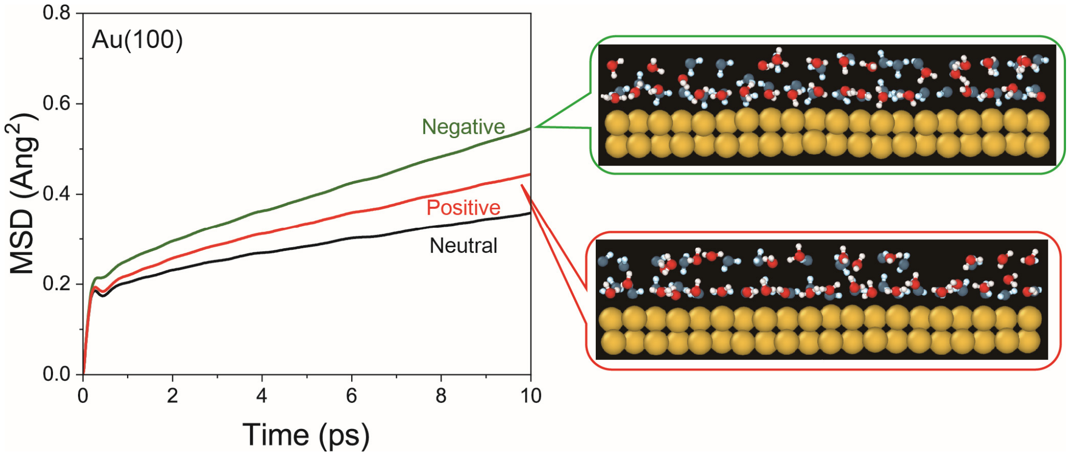

Fig. S11. Potential induced changes in the mobilities of the interfacial water molecules on Au(100). The MSD plot of water molecules within 0.3 nm from the surfaces of negatively charged, neutral, and positively charged Au(100). The side-view snapshots (two atomic rows depth of Au) are overlaid with time-ordered sequence of positions of water molecules at 0 ps (blue) and 1 ps (red) on the negatively and positively charged Au(100) after thermal equilibration of the system.
